# RNA Catalysis, Thermodynamics and the Origin of Life

**DOI:** 10.3390/life4020131

**Published:** 2014-04-10

**Authors:** William G. Scott, Abraham Szöke, Josh Blaustein, Sara M. O’Rourke, Michael P. Robertson

**Affiliations:** 1 Department of Chemistry and Biochemistry, and The Center for the Molecular Biology of RNA, University of California at Santa Cruz, Santa Cruz, CA 95064, USA; E-Mails: sorourke@ucsc.edu (S.M.O.); robertson@chemistry.ucsc.edu (M.P.R.); 2 Lawrence Livermore National Laboratory, Livermore, CA 94551, USA; E-Mail: szoke1@llnl.gov; 3 Department of Chemistry, Cabrillo College, 6500 Soquel Drive, Aptos, CA 95003, USA; E-Mail: josh.blaustein@cabrillo.edu

**Keywords:** ribozymes, RNA self-replication, RNA world, nucleotide 2ʹ,3ʹ-cyclic phosphates, RNA polymerization

## Abstract

The RNA World Hypothesis posits that the first self-replicating molecules were RNAs. RNA self-replicases are, in general, assumed to have employed nucleotide 5ʹ-polyphosphates (or their analogues) as substrates for RNA polymerization. The mechanism by which these substrates might be synthesized with sufficient abundance to supply a growing and evolving population of RNAs is problematic for evolutionary hypotheses because non-enzymatic synthesis and assembly of nucleotide 5ʹ-triphosphates (or other analogously activated phosphodiester species) is inherently difficult. However, nucleotide 2ʹ,3ʹ-cyclic phosphates are also phosphodiesters, and are the natural and abundant products of RNA degradation. These have previously been dismissed as viable substrates for prebiotic RNA synthesis. We propose that the arguments for their dismissal are based on a flawed assumption, and that nucleotide 2ʹ,3ʹ-cyclic phosphates in fact possess several significant, advantageous properties that indeed make them particularly viable substrates for prebiotic RNA synthesis. An RNA World hypothesis based upon the polymerization of nucleotide 2ʹ,3ʹ-cyclic phosphates possesses additional explanatory power in that it accounts for the observed ribozyme “fossil record”, suggests a viable mechanism for substrate transport across lipid vesicle boundaries of primordial proto-cells, circumvents the problems of substrate scarcity and implausible synthetic pathways, provides for a primitive but effective RNA replicase editing mechanism, and definitively explains why RNA, rather than DNA, must have been the original catalyst. Finally, our analysis compels us to propose that a fundamental and universal property that drives the evolution of living systems, as well as pre-biotic replicating molecules (be they composed of RNA or protein), is that they exploit chemical reactions that already possess competing kinetically-preferred and thermodynamically-preferred pathways in a manner that optimizes the balance between the two types of pathways.

## 1. Introduction

Life evolved, according to The RNA World Hypothesis, from prebiotic self-replicating catalytic RNA molecules. This hypothesis was initially formulated by Woese [1], Crick [2] and Orgel [3], but was given significant additional credence and plausibility with the discovery that RNA can be catalytic [4,5]. The RNA World Hypothesis now forms the basis for a wide array of theories of the origin of life [6].

Various mechanisms of pre-biotic RNA polymerization have been proposed; those currently considered to be the most plausible involve condensation of nucleoside 5ʹ-polyphosphates (such as compound B in Scheme I, shown below) in a highly exothermic reaction akin to that catalyzed by modern RNA polymerases, where the departure of a polyphosphate leaving group is critical to the mechanism of RNA synthesis. Liberation of the pyrophosphate, and the concomitant release of dissipative energy or heat, guarantees that the endothermic back reaction will not occur to any significant extent.

**Scheme I:**
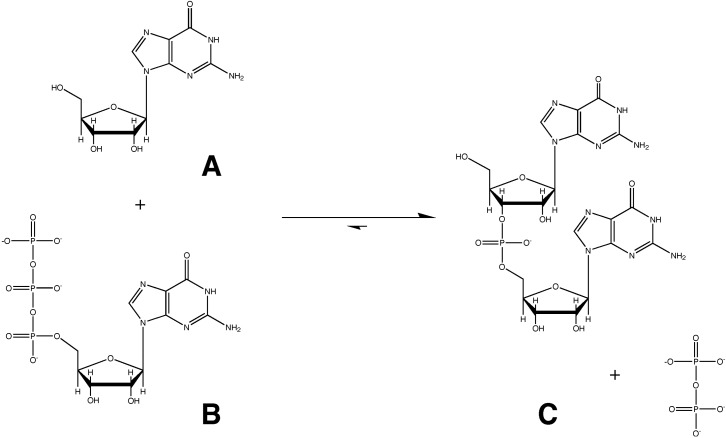
The canonical nucleotide 5ʹ-triphosphate polymerization reaction, employed by all extant polymerases, has a large equilibrium constant (K_eq_ ≈ 10^6^), greatly favoring product formation.

Nucleoside 2ʹ,3ʹ-cyclic phosphates (such as compound D in Scheme II, shown below) are also phosphodiesters. They have, however, been dismissed as alternative potential substrates for pre-biotic RNA polymerization. The reason for this dismissal is the observation that the ΔH_rxn_ (and therefore the ΔG_rxn_) for the forward reaction in Scheme I is large and negative (due to liberation of the pyrophosphate), whereas the ΔH_rxn_ for Scheme II is quite small, since 2ʹ,3ʹ-cyclic phosphodiesters have similar enthalpies to 3ʹ,5ʹ-phosphodiesters (such as compound C). Because of the similar enthalpies (and entropies) of substrate and product [7,8,9,10,11], the forward and back reactions of Scheme II are assumed to occur at similar rates [12,13,14], suggesting that RNA polymerization via this pathway would be much less efficient, as product formation would not be favored in the Scheme II equilibrium.

**Scheme II:**
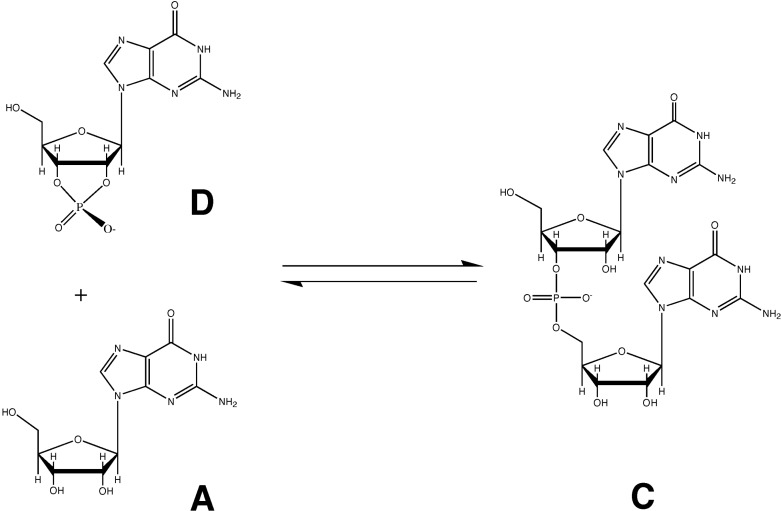
The nucleotide 2’,3’-cyclic phosphate polymerization reaction, typically represented as a simple two-state equilibrium, has an equilibrium constant (K_eq_ ≈ 1) that does not favor product formation.

RNA World Hypotheses therefore usually invoke various pre-biotic syntheses of nucleoside 5ʹ-polyphosphates based upon phosphorimidazolides, phosphorylation reactions involving Pb^2+^ and UO_2_^2+^ ions, followed by nucleotide polymerization catalyzed on adsorption upon active surfaces, or other similarly complex mechanisms [14].

## 2. Formulation of Our Hypothesis

We instead propose that nucleoside 2ʹ,3ʹ-cyclic phosphates are in fact much more plausible candidate substrates for pre-biotic RNA polymerization than has been previously thought, and that they have been dismissed on the basis of a mistaken assumption. Re-examination of this idea has also enabled us to identity a possible driving force of evolution that may help to explain the origin of biological catalysis.

### 2.1. The Previous Assumption

Scheme I is generally accepted as the likely mechanism for RNA synthesis over Scheme II, because an RNA strand synthesized via Scheme II has been assumed to disintegrate via the back reaction at a rate similar to (or greater than) its formation. In other words, an unfavorable equilibrium (positive ΔG_rxn_) has been assumed for Scheme II between an RNA dimer (compound C) and nucleoside 2ʹ,3ʹ-cyclic phosphates (compound D) based on the similar enthalpies of the two types of nucleotide phosphodiesters and the negative ΔS_rxn_ due to adduct formation. (The phosphate is in the diester state in both compounds, and the enthalpic difference due to five-member ring strain is fairly small). If Scheme II in reality represents an equilibrium, then there will be an approximately 50% propensity for each linkage in an RNA polymer formed via Scheme II to disintegrate via the back reaction of Scheme II, so that a stable RNA polymer cannot be maintained (even neglecting the additional contribution of entropy favoring the back reaction). Scheme II has therefore been ruled out as a likely mechanism for RNA synthesis, based upon this equilibrium assumption [14].

### 2.2. Our Proposed Revision

We propose that the above conclusion cannot be correct. In the absence of an enzyme (*i.e.*, a catalyst capable of selectively activating a specific reaction pathway), an RNA dimer (or polymer) synthesized via the forward reaction of Scheme I will be just as susceptible to decay via the Scheme II back reaction as one that is synthesized via the forward reaction of Scheme II. In other words, if we are to invoke the back reaction of Scheme II to dismiss Scheme II as a viable prebiotic synthetic pathway, we are then forced by logical consistency to dismiss Scheme I for exactly the same reasons, since Scheme I and Scheme II produce an identical product. In other words, Scheme II therefore cannot be considered to be a simple, isolated equilibrium; rather Scheme I and Scheme II must be treated as coupled reactions that share a common product. Moreover, since an RNA polymer (synthesized by either scheme) is in fact a relatively stable entity, disintegration via the Scheme II back reaction pathway must somehow be minimized.

To account for the comparatively long lifetimes of structured cellular RNAs such as tRNAs, rRNAs, ribozymes and mRNAs, we must treat the system as two coupled, three-state reactions. One of these three-state reactions (a revision of Scheme II) is shown below as Scheme III (where the additional equilibrium between C and Cʹ is appended to Scheme II as shown, as well as to Scheme I in an analogous manner). By doing so, we can then avoid this logical contradiction.

When this third state (Cʹ) is also taken into account, the basis for dismissing Scheme II (or Scheme III) as a viable pre-biotic RNA synthesis pathway, weakens considerably. RNA polymers survive for a reasonably extended period of time because they become trapped in a local potential energy minimum corresponding to Cʹ. RNA in the Cʹ state is a kinetically favored product even though it is not necessarily a thermodynamically favored product; nucleotide stacking interactions, as well as base-pairing, drives RNA helix formation. Helical base-stacking in particular, greatly stabilizes the Cʹ product relative to C via a large favorable entropic contribution; whereas, extensive complementary base-pairing hydrogen bonding interactions increase the energy barrier between Cʹ and C, thus decreasing the rate of helical melting. Helical formation of the RNA polymer (Cʹ) traps the phosphodiester backbone of the RNA in the anti-periplanar (g-g-) phosphodiester conformation [15] that prevents in-line attack of a 2ʹ-OH upon an adjacent phosphodiester linkage, thus inhibiting the phosphodiester isomerization back-reaction that requires an S_N_2(P) geometry (Scheme II and left-hand side of Scheme III). The net effect of this, from a thermodynamics point of view, is exactly analogous to precipitation of the product; sequestering compound C (the more labile conformation of RNA in which the phosphate resembles the transition-state geometry) by locking it into the less labile helical conformation, Cʹ, drives the RNA polymerization reaction toward product formation, regardless of whether it proceeds via Scheme I or Scheme II. The RNA polymer is thus preserved by kinetically trapping the polymerized product in a stable, helical conformation that cannot readily access the transition-state geometry required for the decomposition reaction to take place.

**Scheme III:**
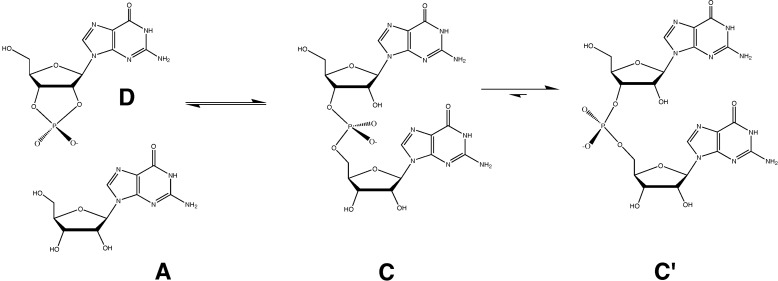
We propose replacing the simple two-state equilibrium depicted in Scheme II with a three-state equilibrium, where Cʹ represents helical stabilization of the product, effectively suppressing the back reaction.

We further hypothesize that a fundamental property of living systems as well as pre-biotic replicating molecules is that they are able to optimize the balance between kinetically-favored and thermodynamically-favored biochemical pathways in such a manner that the probability of their growth and replication becomes maximized. Enzymes, whether self-replicating RNAs, other RNA catalysts, or protein enzymes, have thus evolved as the means for doing this most efficiently. Optimization of the balance between kinetically-favored and thermodynamically-favored biochemical pathways via replication coupled to natural selection is thus likely to be a major driving force behind the evolution of enzyme catalysis and life. We will return to this point below.

## 3. Results and Discussion

### 3.1. Pre-Biotic RNA Synthesis with Nucleotide 2ʹ,3ʹ-Cyclic Phosphates is in Fact Plausible

We have argued that the assumed equal partition between reactants and products in Scheme II cannot be used to rule out this pathway in favor of Scheme I. This does not, however, prove that pre-biotic RNA synthesis took place via Scheme II rather than Scheme I. Scheme I is unambiguously favored completely over Scheme II by modern organisms that can readily synthesize nucleotide 5ʹ-triphosphates, and evolution very clearly has favored Scheme I as the unique mechanism of nucleic acid biosynthesis. Pre-biotic RNA synthesis via Scheme II, however, provides some significant biochemical advantages in a pre-biotic milieu and possesses the additional merit of significant explanatory power with respect to biochemical evolution. Six such examples of the explanatory power of this hypothesis are described as follows:

#### 3.1.1. The Fossil Record (Ribozymes)

Ribozymes are thought to be, in some sense, molecular fossils. Peptidyl-transferase, for example, likely evolved quite early in biological history, and its preservation in the form of a ribozyme catalyst, as well as its fragility with respect to evolutionary tinkering, is clear and compelling evidence for its ancient origins [16]. At least half of the naturally-occurring modern-day ribozymes (including the hammerhead, hairpin, HDV and VS RNAs) catalyze the Scheme II reaction, and two others catalyze other phosphodiester isomerizations [17]. No known, naturally-occurring, ribozyme catalyzes the Scheme I reaction [18].

If these modern-day ribozymes are also molecular fossils, it would seem reasonable that the Scheme II reaction, which is the simplest and most ubiquitous reaction catalyzed by RNA, has rather ancient origins. It is therefore quite possible that the chemistry of cleavage and ligation employed by these isomerase ribozymes is evolutionarily derived from a more general RNA-catalyzed RNA polymerization reaction that occurred via a mechanism essentially identical to the ribozyme-catalyzed ligation reaction, apart from being dependent upon a mobile template. If so, this polymerization mechanism eventually would have been superseded by Scheme I-type polymerizations once other enzymes had evolved to supply 5ʹ-polyphosphate substrates in large abundance.

#### 3.1.2. Comparative Abundance of Nucleotide 2ʹ,3ʹ-Cyclic Phosphates

The efficiency of prebiotic RNA self-replication would likely have been limited by the availability of nucleotide substrates. Because nucleoside 2ʹ,3ʹ-cyclic phosphates are comparatively simple to synthesize, and because proto-cellular membranes would likely be much more permeable to the singly-charged nucleotide 2ʹ,3ʹ-cyclic phosphates than they would be to nucleotide polyphosphates having multiple charges, it is likely that nucleoside 2ʹ,3ʹ-cyclic phosphate substrates would be relatively more plentiful and available in higher concentrations for prebiotic RNA synthesis. In addition, since the natural decomposition products of RNA are nucleoside 2ʹ,3ʹ-cyclic phosphates, the starting reagents for pre-biotic RNA synthesis are continually recycled and would likely remain in abundance in a prebiotic environment, limited only by the comparatively slower hydrolysis of the 2ʹ,3ʹ-cyclic phosphate. Nucleoside 5ʹ-polyphosphates cannot be recycled in this way. Instead, nucleoside 5ʹ-phosphates must be regenerated each time they are to be incorporated in a new strand of RNA, in an energetically costly (and therefore, in the absence of a catalyst, improbable) manner.

#### 3.1.3. Nucleotide 2ʹ,3ʹ-Cyclic Phosphates and Prebiotic Pyrimidine Nucleotide Synthesis

Although purine and pyrimidine nucleotide bases are fairly straightforward to synthesize chemically under plausible pre-biotic conditions, the problem of how these might condense upon ribose to form ribonucleotide adducts is much more challenging. Purine ribonucleotides form inefficiently [19], and pyrimidine ribonucleotides will not form at all [20]. An alternative plausible pre-biotic synthetic pathway for pyrimidine ribonucleotides has recently been demonstrated [21]. Remarkably, the final step of the synthesis involves the addition of phosphoric acid to the immediate precursor to generate the ribonucleotide. The phosphate adds to what will become the 3ʹ-oxygen and 2ʹ-carbon to produce a ribonucleotide 2ʹ,3ʹ-cyclic phosphate possessing the correct stereochemistry (*cf*. Figure 1 in [21]). If 2ʹ,3ʹ-cyclic phosphate ribonucleotides were the original prebiotic substrates for RNA polymerization, this synthetic pathway immediately suggests how these substrates might arise.

#### 3.1.4. Simplicity of the Reaction would Allow Evolutionary Boot-Strapping

Nucleoside 2ʹ,3ʹ-cyclic phosphate isomerization (Scheme II) is arguably the simplest chemical reaction involving RNA, and it occurs to an appreciable level at neutral pH in pure water, especially in unstructured RNA. No divalent metal ions, *ad hoc* heating and cooling cycles, or heterogeneous surfaces are required for catalysis; this is well-known to be the case in RNase A catalyzed reactions as well as the non-catalyzed reaction. Recently, we have demonstrated that three of the four small self-cleaving ribozymes listed above that utilize this pathway also do not require divalent metal ions for catalysis [22]. Only the RNA itself (along with any counter-cations, even non-metallic monovalent cations) is required to catalyze the hammerhead, hairpin and VS ribozyme reactions, and therefore it is likely that an ancient RNA-based polymerase that used nucleotide 2ʹ,3ʹ-cyclic phosphate substrates would not have to depend on scarce metal ions as cofactors either. Because nucleoside 2ʹ,3ʹ-cyclic phosphates are much more straightforward to synthesize than nucleoside 5ʹ-polyphosphates, no specially-modified phosphorimidazolides or other activated precursors need to be invoked to explain how starting materials might be supplied. Once enzymes capable of synthesizing nucleoside 5ʹ-phosphates have evolved, nucleoside 5ʹ-phosphates could be produced in sufficient abundance to enable enzymatic synthesis via Scheme I to evolve. In other words, RNA synthesis via Scheme II may have provided a convenient evolutionary boot-strapping mechanism to enable efficient synthesis via Scheme I to evolve to take its place.

#### 3.1.5. A Viable First RNA Editing Mechanism

The product (dimerized or polymerized RNA) may be thought of as a two-state system, in which the initial state that develops instantaneously (compound C) as a result of dimerization is in an approximate 1:1 equilibrium with reactants. Upon nucleotide base-pairing, the initial state then adopts the helical form of the final state (compound Cʹ) that becomes kinetically trapped and therefore inaccessible to the back reaction. Promotion of the initial product state (C) to the final helical product state (Cʹ) depends upon correct base-pairing; an improper fit impedes helical formation and thus increases the propensity for the back-reaction to occur. This increased propensity for the back reaction facilitates elimination of incorrectly-paired nucleotides. Helical editing and fidelity of replication thus arise in a completely natural manner. Hence, a primitive editing mechanism based upon Scheme II is much more energetically plausible than a primitive editing mechanism based upon Scheme I. Modern polymerases of course use an editing mechanism based upon Scheme I, but they most certainly possess much richer functionality that a pre-biotic RNA polymerase would be likely to possess.

#### 3.1.6. The Advantage of RNA *vs*. DNA

The idea that the first self-replicating nucleic acid must have been RNA rather than DNA was based originally on the assumption that DNA lacked the ability to be catalytic; no naturally-occurring deoxyribozymes have yet been discovered. However, using *in vitro* selection techniques, DNA has been shown to possess the ability to catalyze phosphodiester isomerization reactions as well [23]. Nature ultimately preferred DNA to RNA as the molecule best suited to storing and replicating genetic information. If DNA can also be catalytic, why propose an RNA world rather than a DNA world? The main difference between RNA and DNA is the presence of a 2ʹ-OH in RNA. Originally, this was thought to be critical to the formation of tertiary structures having sufficient complexity to form an enzyme structure. However, the crystal structures of several ribozymes reveal a fairly minimal reliance upon 2ʹ-OH-mediated hydrogen bonds, and the existence of *in vitro* selected deoxyribozymes refutes the absolute necessity of having 2ʹ-OH in a nucleic acid enzyme structure. The remaining advantage of the 2ʹ-OH is that it alone allows formation of 2ʹ,3ʹ-cyclic phosphates. RNA thus possesses an advantage over DNA as a candidate for the original pre-biotic self-replicating polymer in that its constituents can be either nucleotide 5ʹ-polyphosphates, that can be assembled according to Scheme I, in analogy to DNA polymerization, or 2ʹ,3ʹ-cyclic phosphates, that can be assembled via a more primitive route, according to Scheme II. In contrast, if Scheme I represents the original pre-biotic nucleic acid replication mechanism, there is little reason to favor RNA substrates over DNA (or a mixture of the two types of nucleotide substrates).

## 4. Conclusions

### 4.1. The Origin of Catalysis and the Origin of Life

Erwin Schrödinger, in his seminal book “What is Life?” [24] proposed that a characteristic physical property of living organisms is that they feed on “negentropy” and thus appear to sidestep the second law of thermodynamics. This explanation requires the *ad hoc* introduction of a new, biologically emergent, thermodynamic quantity that has never been measured. Although this idea has never been reconciled with the second law of thermodynamics, the idea that living organisms are unique in their ability to generate and replicate ordered systems from disordered components certainly appears to be sound.

Returning to our claim that enzymes have evolved to optimize the balance between kinetically-favored and thermodynamically-favored biochemical pathways, we can begin to understand Schrödinger’s problem without resorting to his *ad hoc* invention of a new thermodynamic potential. Instead, we can reformulate Schrödinger’s claim as follows: A fundamental property of living systems as well as evolving pre-biotic replicating molecules is that they exploit chemical reactions that already possess both a kinetically-preferred pathway and a thermodynamically-preferred pathway, and they do this in such a way that optimizes the balance between the two. The optimization process is an evolutionary one, and optimization of the balance can be defined loosely but operationally as changing the propensities of the two pathways relative to one another in successive rounds of replication and natural selection in such a way that maximizes the ability of the system to grow and to replicate itself with reasonable fidelity.

It has always been recognized that enzymes have evolved to accelerate the rate of biochemical reactions that benefit an organism. We suggest that an additional selective restraint placed upon enzymes by the evolutionary process is the ability to store information and energy in metastable forms that can then be exploited by the organism or pre-biotic replicating molecule. Kinetically-favored but thermodynamically less favored reaction products, such as RNA polymers, are perhaps the original example, and certainly one of the most important examples, of this principle. However, this principle may be generalized to include much, if not all, of metabolic biochemistry, photosynthesis, and bio-energetics. The description of this principle in its most general form is the subject of a previous communication [25].

### 4.2. Implications for the in Vitro Evolution of an RNA Self-Replicase

The Holy Grail of RNA *in vitro* evolution experiments is to discover a sequence of RNA capable of RNA polymerization or even self-replication. Although such an experiment, in the absence of time-travel, cannot prove that the RNA World Hypothesis is correct, successful generation of an RNA replicase would constitute a compelling proof of principle.

*In vitro* RNA evolution experiments aimed toward evolving RNA ligases and polymerases have focused on substrates having 5ʹ-triphosphates. Although these have met with significant success, it would be of interest to know whether *in vitro* evolved ribozymes that use 2ʹ,3ʹ-cyclic phosphates, like their naturally occurring counterparts, might arise more readily or otherwise possess additional assets that might facilitate *in vitro* selection of a polymerizing or replicating ribozyme, due to the comparative simplicity of the Scheme II reaction compared to that of Scheme I. Our research group is currently testing this hypothesis and will publish results that support this idea in a forthcoming communication. In addition, a newly available report describes a ribozyme derived from the naturally-occurring hairpin ribozyme that catalyzes the addition of all four nucleotide 2ʹ,3ʹ-cyclic phosphates to the 5ʹ-hydroxyl termini of RNA to form a 3ʹ to 5ʹ phosphodiester product, in accordance with what has been proposed here [26].
